# QTest: A new way to easily sample, store, and ship samples to perform Q fever PCR analysis on bulk tank milk

**DOI:** 10.3168/jdsc.2021-0144

**Published:** 2021-09-23

**Authors:** Michael Treilles, Pierre Charollais, Raphaël Guatteo, Carla Azevedo, Damien Achard, Juan Munoz-Bielsa, Philippe Gisbert

**Affiliations:** 1 Laboratoire Qualyse, ZAE Montplaisir, 79220 Champdeniers-St. Denis, France; 2 INRAE, Oniris, BIOEPAR, 44300 Nantes, France; 3 Ceva Santé Animale, 10 avenue de La Ballastière, Libourne 33500, France

## Abstract

Bulk tank milk (BTM) is an easy, inexpensive, and representative sample for detection of *Coxiella burnetii* infections (Q fever) in dairy herds using real-time PCR. Bulk tank milk PCR can be performed either for initial herd screening or for monitoring the effectiveness of preventive measures. However, one major limitation under field conditions is the need to deliver BTM samples in adequate condition (quickly, safely, and under refrigeration) to a qualified laboratory. In addition, sending non-inactivated biological samples via normal mail may be prohibited. We developed an innovative, easy, and accurate diagnostic tool (QTest) for Q fever to support veterinarians and farmers in overcoming these constraints. The farmer or veterinarian simply places some drops of BTM on a Whatman FTA Elute Micro Card (FTA card) and lets the card dry before mailing it to the laboratory. In a 2-step study, we tested the hypotheses that (1) BTM samples stored on FTA cards are stable over time and at different temperatures, and (2) PCR results obtained via FTA cards are consistent with those obtained from raw BTM samples. The stability of *C. burnetii* DNA in milk preserved on an FTA card was maintained for at least 29 d at room temperature or 37°C to mimic field conditions. In our field study, of the original 70 positive BTM samples (when tested on raw BTM just after sampling), 58 samples were positive (on either raw BTM or FTA card) by the time of the direct comparison study (10 to 14 d later). Of these 58 samples, 45 raw BTM samples still tested positive after aging, and 53 FTA card BTM samples tested positive, indicating that detection was higher using FTA cards (91.4%) than raw milk (77.6%). Therefore, with inactivation and shipping advantages, this technique facilitates an easier and more practical approach to diagnosis of Q fever at the herd level and would support Q fever control strategies, especially in countries lacking adequate and close laboratory facilities.

## Highlights

• Bulk tank milk is an easy and cheap matrix in which to detect Q fever in dairy herds.
• The diagnosis of Q fever is possible on bulk tank milk using FTA cards.
• QTest allows the storage of bulk tank milk in field conditions for at least 29 days.
• By inactivating Coxiella burnetii, FTA cards avoid any zoonotic risk of Q fever.
• QTest makes Q fever diagnosis easier by avoiding storage and shipping constraints.

## Graphical Abstract

**Figure:**
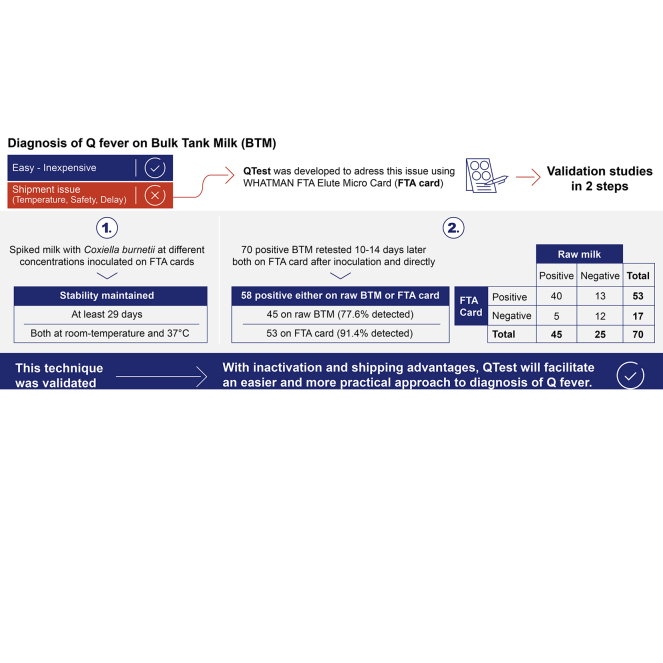
**Summary:** In dairy cattle at the herd level, *Coxiella burnetii* can be diagnosed by PCR on bulk tank milk (BTM). To facilitate the collection and shipment of BTM samples to the laboratory, we validated the use of Whatman FTA Elute Micro Cards for the conservation of BTM in 2 studies. The first study evaluated the stability of samples over time and at 2 different storage temperatures. The second compared results for BTM stored on FTA cards and raw BTM stored as is.

Q fever is a zoonotic disease caused by an obligate intracellular bacterium, *Coxiella burnetii* (Arricau-Bouvery and Rodolakis, 2005), with ruminants being considered the main reservoir for human infection. This disease, described for the first time among abattoir workers in Australia, is now recognized as being endemic worldwide (Maurin and Raoult, 1999). Based on available data, the average prevalence at the animal level is between 15 and 30% for cattle, sheep, and goats (Guatteo et al., 2011), with large variability between countries. For example, a French study (Gache et al., 2017) reported seroprevalence at herd levels of 36, 56, and 61% in cattle, sheep, and goats, respectively. However, because of frequent asymptomatic carriers, nonspecific clinical signs, and a challenging diagnosis, only a small percentage of farmers and veterinarians know that *C. burnetii* infection is present on their farms. *Coxiella burnetii* infection in cattle has been associated with reproductive disorders such as abortion, stillbirth, and weak newborns (Agerholm, 2013), retained fetal membranes (López-Gatius et al., 2012), endometritis (De Biase et al., 2018), and infertility (Lopez-Helguera et al., 2014). The multifactorial origin of reproduction disorders and the frequent asymptomatic pattern of Q fever in ruminants has frequently led to underestimating the implication or the circulation of *C. burnetii* in cattle herds.

Shedding of *C. burnetii* differs among ruminant species, with milk being the most frequent shedding route in cattle (Rodolakis et al., 2007). Milk is also an easy and cheap sample to collect. Therefore, bulk tank milk (**BTM**) is currently used to detect the presence of *C. burnetii* infection in herds, typically using real-time PCR (**RT-PCR**) analysis (Guatteo et al., 2007a). For example, during the Q fever outbreak in the Netherlands, monitoring of infected sheep and goat farms was performed using PCR applied on BTM samples (van den Brom et al., 2012). Indeed, BTM samples are suitable for repeated sampling. Thus, RT-PCR analysis of BTM samples is frequently used in dairy herds to evaluate the presence of *C. burnetii*, either for initial herd screening or for monitoring the effectiveness of preventive health schemes (Guatteo et al., 2007a; Taurel et al., 2012, 2014; Valergakis et al., 2012; Piñero et al., 2014).

Nevertheless, one important limitation for large-scale implementation of this testing method is the need to deliver the BTM samples in well-preserved condition (within 24 h and refrigerated) and safely to a veterinary diagnostic laboratory having expertise and facilities suitable to perform Q fever testing using RT-PCR. Unfortunately, this is not always achievable under field conditions, particularly while respecting the zoonotic potential of *C. burnetii*. In addition, sending non-inactivated samples for diagnosis by road or air is constrained by regulation and may be prohibited in some countries.

With the aim of raising awareness about this dangerous but preventable disease, we have validated a new diagnostic tool (**QTest**) for Q fever to support veterinarians and farmers with an easy and accurate qualitative diagnostic solution. QTest is very simple to use: the farmer or veterinarian collects a small amount of BTM and places it on a Whatman FTA Elute Micro Card (**FTA card**). FTA cards are cellulose paper cards containing chemicals that lyse cells and denature proteins while maintaining DNA integrity, theoretically ensuring preservation of the BTM sample for DNA detection over time. Samples collected on FTA cards are suitable for molecular detection of pathogens with minimal contamination risk and without the need for fixative agents or refrigeration for preservation. This makes it possible to send samples safely via conventional mail or courier without the need to refrigerate.

FTA cards have been previously used for the detection of other bacteria in BTM samples (Durel et al., 2015; Azevedo et al., 2016) and with other matrices, such as blood, for detection of *C. burnetii* (Pascucci et al., 2015) in wild animals. To our knowledge, FTA cards have never been used for *C. burnetii* DNA detection from BTM. This tool offers a breakthrough innovation to farmers and veterinarians, increasing awareness of the disease and leading to better management of the disease in herds as well as better zoonotic risk management.

This study had 2 different but complementary objectives. The first was to evaluate the preservation of BTM samples for *C. burnetii* DNA detection on an FTA card over time (d 1 to 29) under 2 temperatures [room temperature (20–22°C) and 37°C] to mimic field preservation and transport conditions and at different *C. burnetii* concentrations. The second objective was, in a field study, to compare the detection of *C. burnetii* DNA after aging either when RT-PCR was applied directly on raw BTM samples or on BTM preserved on FTA cards.

In the first study, raw BTM known to be free of *C. burnetii* DNA was spiked with *C. burnetii* to reach a load of ~5 × 10^6^ genome equivalents (GE)/mL, which became the master sample. Using the original *C. burnetii* DNA-free raw BTM, the master sample was then diluted to achieve dilutions of 10^−1^, 10^−2^, 10^−3^, 10^−4^, and 10^−5^. Then, 200 µL of each dilution was deposited on each of 4 dedicated spots on 4 FTA cards (i.e., 16 spots per dilution), to ensure a sufficient number of replicates to allow testing at 2 temperatures and several sample testing times. The FTA cards were allowed to dry at room temperature (20–22°C) for 1 h (on d 0) to mimic field conditions. Half of the cards were stored at room temperature and half were stored at 37°C (to mimic storage and transport under field conditions). To assess the effect of duration of storage on the ability of the dried milk spots (**DMS**) to preserve *C. burnetii* DNA, within each temperature, each dilution was tested using RT-PCR at different times (d 1, 4, 6, 8, 11, 15, 20, and 29).

On each of these 8 days, DNA extraction was performed directly from FTA cards (room temperature and 37°C) as described by Tardy et al. (2019). Briefly, each DMS was carefully excised and placed in bead beating tubes (Lysing Matrix E 2-mL tubes, MP Biomedicals) containing 1.4-mm ceramic spheres, 0.1-mm silica spheres, and a single 4-mm glass bead. After adding 1.5 mL of molecular biology–grade water, the tubes were shaken for 40 s at 6,000 rpm in a FastPrep homogenizer (MP Biomedicals). They were then incubated at 100°C for 20 min and centrifuged for 1 min at 10,000 × *g* at room temperature. Each nucleic acid extract was preserved at −20°C until the day of the common RT-PCR amplification (performed on d 32). Common amplification of all extracts in a single run was performed to avoid assay bias. Amplification of 5 µL of each extract was then performed using a commercial Q Fever PCR amplification kit (VetMAX *C. burnetii* Absolute Quant Kit, Thermo Fisher Scientific). This kit provides a real-time TaqMan PCR amplification, targeting *IS1111* and using an endogenous internal positive control (*GAPDH*) to ensure the quality of the amplification process. The RT-PCR was performed using a CFX96 Touch Real-Time PCR Detection System (Bio-Rad).

To assess RT-PCR data from FTA cards, the obtained cycle threshold (**Ct**) values were analyzed using ANOVA, with dilution, duration of storage (d), and temperature as factors of variation. Pair-wise comparisons were performed using the Tukey test.

For undiluted milk, the Ct was higher for FTA cards than for raw milk. Specifically, the elution of 200 µL of raw milk required 100 µL of buffer solution whereas the elution of 200 µL of milk trapped on a DMS on an FTA card required 1,500 µL of buffer solution. Thus, for the same starting sample, the test sample for the extraction step was 15 times more diluted in the sample coming from an FTA card than in the sample coming from raw milk. This factor of 15 is approximately equal to 2^4^ (16); therefore, from a mathematical point of view, a difference of 4 Ct between raw milk and FTA card elutions is expected.

Regardless of the duration of storage of the DMS, all samples undiluted or diluted between 10^−1^ and 10^−3^ were detected to contain *C. burnetii* DNA. No significant loss of detectability (i.e., analytic sensitivity) was noted from d 1 to 29, regardless of dilution or storage temperature. This means that the FTA card system ensures stable preservation of *C. burnetii* DNA in samples stored at room temperature or 37°C for at least 29 d. Duration of storage had no effect on *C. burnetii* DNA detection (Table 1), except for dilution 10^−3^; this difference was due only to the Ct value on d 11; Ct values on d 15, 20, and 29 did not indicate a decrease in sensitivity. Temperature had a significant effect for the undiluted and 10^−1^ dilution samples. Nevertheless, at these levels of dilution, the Ct values (all <30) indicated that there was no risk of not detecting a positive sample. For more diluted samples, no significant effect of storage temperature was observed. Therefore, we can assume that duration of storage and temperature had no effect on the limit of detection when BTM was preserved on FTA cards (Table 1).

**Table 1:** Cycle threshold (Ct) values for *Coxiella burnetii* DNA detection after real-time PCR of dried milk spots on Whatman FTA Elute Micro Cards (FTA) after storage at room temperature (20–22°C) or 37°C for up to 29 d

Time	Master sample	Tested samples											
		Undiluted		Dilution 10^−1^		Dilution 10^minus;2^		Dilution 10^minus;3^		Dilution 10^minus;4^		Dilution 10^minus;5^	
		20–22°C	37°C	20–22°C	37°C	20–22°C	37°C	20–22°C	37°C	20–22°C	37°C	20–22°C	37°C
Day 0	20.8												
Day 1		26.0^a^	22.8^b^	27.2^a^	27.3^b^	30.7	30.3	33.1	33.1	38.7	ND^2^	ND	ND
Day 4		24.2^a^	21.6^b^	26.6^a^	25.5^b^	28.4	31.2	32.2*	31.2*	ND	39.1	ND	ND
Day 6		24.3^a^	22.8^b^	27.6^a^	26.3^b^	29.1	30.1	32.4	32.3	34.5	37.4	ND	38.2
Day 8		24.4^a^	22.0^b^	27.1^a^	27.3^b^	28.1	29.3	32.7	31.5	36.1	ND	ND	ND
Day 11		24.6^a^	21.4^b^	26.2^a^	26.4^b^	29.1	29.7	34.4*	33.8*	36.0	38.0	ND	ND
Day 15		25.2^a^	21.4^b^	27.3^a^	25.4^b^	29.3	29.8	33.0	32.7	ND	37.9	ND	ND
Day 20		25.2^a^	22.4^b^	27.2^a^	24.5^b^	29.6	29.8	31.7	32.7	ND	38.4	ND	ND
Day 29		23.4^a^	23.1^b^	26.8^a^	26.2^b^	30.5	30.1	33.5	32.2	34.4	36.8	ND	ND
													
Mean^1^		24.7^a^	22.2^b^	27.0^a^	26.1^b^	29.4	30.0	32.9	32.4				
SD^1^		0.81	0.69	0.43	0.97	0.92	0.55	0.83	0.84				
*P*-value (time)^1^		0.635		0.517		0.408		0.046					
*P*-value (temperature)^1^		0.001		0.049		0.100		0.129					

a,b For nondiluted and dilution 10^−1^ samples: within each dilution, the values do not differ significantly with time (*P* > 0.05) but differ significantly with temperature (*P* < 0.05).

1 As *Coxiella burnetii* DNA was not detected at all times and temperatures, the mean, SD, *P*-value (time), and *P*-value (temperature) were not calculated.

2 Not detected.

* Values on d 4 differed (*P* < 0.05) from those on d 11.

In the second study (the field-based trial), 414 randomly selected BTM samples from western France were provided by a dairy farm advisory laboratory (LILCO Surgères, France) to obtain at least 60 BTM samples positive for *C. burnetii* DNA (on the assumption that the prevalence of positive BTM would be around 15%).

The majority of farms used in the study had tanks equipped with programmed agitation (2 min every 13 min) but, in addition, the sampler stirred the milk for at least 1 min to ensure it was homogeneous. At least 45 mL of BTM was collected in a preservative-free (i.e., no bronopol) vial. The traceability of the BTM samples was ensured by the samplers by using barcode labels. The vials were stored at 0 to 4°C and sent to LILCO within 24 h of sampling. Samples arrived at our RT-PCR laboratory 3 d after sampling and were processed on the same day, except for 10 samples that were processed 8 d after sampling for technical reasons (Figure 1).

**Figure 1:**
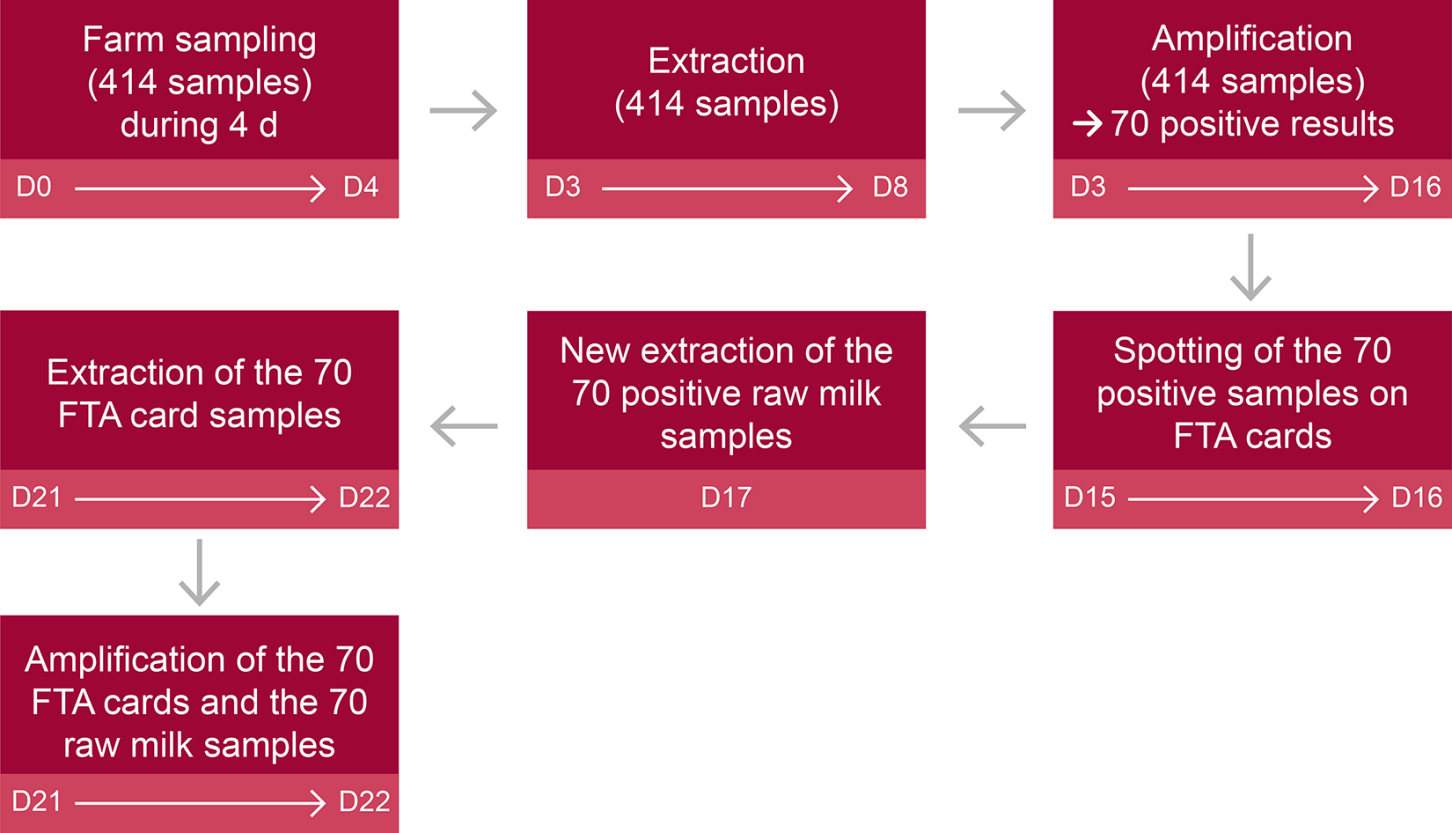
Sample workflow for the second (field trial) study (FTA cards: Whatman FTA Elute Micro Card). D = day.

After storage for up to 29 d, to compare the detection of *C. burnetii* DNA using RT-PCR either directly from raw BTM or from DMS on FTA cards, the 70 BTM samples known to be PCR positive 3 d after sampling were spotted at d 15 to 16 on FTA cards (200 µL in each spot) to dry at room temperature. To simulate a typical period of shipping, the DMS on FTA cards were stored at room temperature for 1 wk before beginning the extraction process.

The extractions and RT-PCR were performed as described for the first study. Specifically, 200 µL of raw milk was mixed with 180 µL of lysing buffer and 25 µL of proteinase K. Incubation was performed for 30 min at 70°C. Extraction was performed using a Thermo Scientific KingFisher Flex Magnetic Particle Processor with up to 96 extractions in a single run.

The DNA extracts were preserved at −20°C until amplification was performed. Amplification was not possible in a single run (BTM samples × 70 and DMS samples × 70), so it was performed in 2 batches 2 d apart. However, within each batch, pairs of 35 BTM and 35 DMS samples were tested to avoid any effect of day of amplification or batch on results. In addition, the same kit, equipment, and technician were used on both days. The analyses were carried out under good laboratory practice and quality assurance procedures. In addition, a standard positive control sample was monitored using a control chart. As described previously, a housekeeping gene (*GAPDH*) was also amplified in each well.

Agreement between qualitative results of both variables (DMS on FTA card/raw milk) was evaluated using Cohen's kappa (κ) coefficient. Positive and negative counts were analyzed using a generalized linear model with a binomial distribution and a log link function, with method (DMS on FTA card/raw milk) as a covariate. Comparisons were performed using a normal approximation based on LSM estimates and standard errors (SE) derived from the model.

We found 70 positive BTM samples (at d 3 after sampling) out of 414 tested, indicating an apparent prevalence of 16.9% for detection of *C. burnetii* DNA in those herds. Although samples were preserved at 4°C, when we retested the 70 BTM raw samples 10 to 14 d later (when the DMS were spotted and tested), only 45 were still positive when performing extraction and PCR from raw but aged BTM. Therefore, the rate of detection for an aged BTM sample can be estimated at 64.3% compared with that for fresh BTM. Furthermore, for the samples (n = 45) that were still positive in the second analysis, the average Ct value was 1.6 higher than in the first analysis. This translates to an approximate 3-fold (2^1.6^ = 3.04) decrease in *C. burnetii* DNA load over the period from 10 to 14 d at 4°C. This clearly indicates the need for a better method (i.e., FTA cards) to preserve *C. burnetii* in milk samples, especially when the delay between sampling and RT-PCR analysis cannot be reduced.

Of the original 70 samples that were positive when tested fresh, 58 samples tested positive using one or both of the storage/transport options by the time of the direct comparison study 10 to 14 d later. Of these 58 samples, 45 raw BTM samples tested positive and, of these, 5 tested negative when using DMS on FTA cards (in graphical abstract and Figure 2). In other words, we had 13 false-negative results with older raw BTM samples but only 5 false-negative results for older DMS on FTA cards. These 5 remaining samples were all weak positive (Ct >35). In conformity with PCR theory, their non-detection was likely due to the lack of reproducibility of weak positive samples.

**Figure 2:**
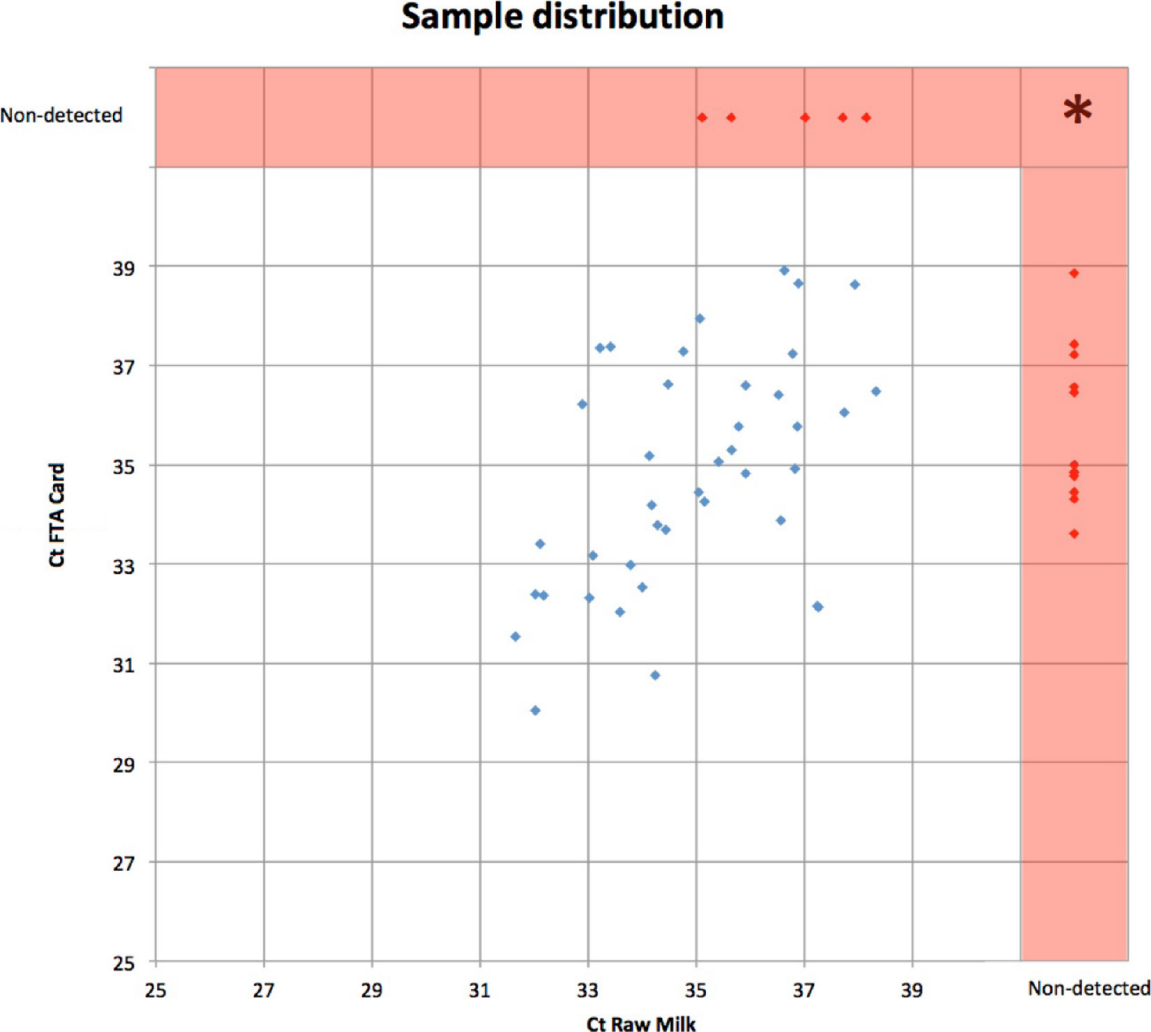
The distribution of positive (blue dots) and negative (red dots) PCR results (expressed in cycle threshold values, Ct) for *Coxiella burnetii* DNA detection in raw milk samples and in dried milk spots on Whatman FTA Elute Micro Cards (FTA cards). *Represents 12 samples that were negative for both sample types (raw milk and FTA cards).

Considering the 70 positive samples, we found a Cohen's κ of 0.40, indicating only fair agreement between techniques. This low agreement can be explained by the lack of reproducibility of weak positive samples, which was confirmed by a perfect κ value of 1 when considering only the raw milk samples with Ct <35, corresponding to reproducible positive results.

Importantly, for 13 samples, FTA cards produced positive PCR results whereas the equivalent raw BTM samples tested gave negative PCR results (Figure 2). This indicates that detection was higher using FTA cards with aged BTM (91.4%) than with raw aged BTM (77.6%) samples. The probability of having a false-negative result was 2.6 times (95% CI: 0.99; 6.82) less when using FTA cards compared with raw milk (*P* = 0.05).

Because *C. burnetii* is prevalent in dairy herds worldwide (Valergakis et al., 2012) and can lead to detrimental effects (at clinical or subclinical levels) in herds (López-Gatius et al., 2012), it is clearly advantageous for farmers to know if *C. burnetii* is circulating within their herds. As well as causing issues in farm and wild animals, Q fever can adversely affect humans (Roest et al., 2011).

As *C. burnetii* shedding into milk is routine, BTM samples are an ideal method of sampling for *C. burnetii* but, unfortunately, raw BTM samples are susceptible to degradation during transportation. This study also shows that raw milk is sensitive to aging.

Based on our findings, we conclude that *C. burnetii* DNA detection based on DMS on FTA cards is effective and can be considered an excellent alternative to raw BTM samples, given the ability of FTA cards to preserve the sample regardless of the temperature and transportation or storage time. This option can prevent the problems encountered under field conditions; indeed, FTA cards are already used for the diagnosis of pathogens responsible for mastitis in milk, including *Staphylococcus aureus*, *Streptococcus agalactiae*, and *Mycoplasma* spp. (Durel et al., 2015; Tardy et al., 2019).

Importantly, we detected no significant effect of storage duration of the FTA cards on *C. burnetii* DNA detection. The only effect of temperature was noted in the undiluted and lowest dilution samples, where storage at 37°C yielded lower Ct values than did storage at room temperature. We can speculate that chemical activity was enhanced at the higher temperature. In the field study, we detected an effect of duration of storage for raw BTM samples: only 45 of the original 70 positive samples remained positive after storage for 10 to 14 d at 4°C. This supports the need for an alternative storage and transportation option for *C. burnetii* analysis in BTM samples.

Guatteo et al. (2007b) assessed the risk for false-negative results when *C. burnetii* DNA detection by RT-PCR test was delayed after the time of sampling; they found that only about two-thirds of positive samples remained positive after 5 d at 4°C or after 63 d at −20°C, but they also noted that persistently positive samples had higher initial loads of *C. burnetii* DNA. Thus, the risk for false-negative results remains high for samples with low or moderate *C. burnetii* DNA loads when analysis is delayed after sampling.

To date, when using QTest, the laboratory results were expressed as either positive (detected) or negative (not detected). Further studies should be conducted to define relevant semiquantitative classes. Furthermore, a negative laboratory diagnosis must be interpreted in association with epidemiological and clinical data because there is the possibility of false-negative results for the QTest in cases where there is a low load of *C. burnetii* DNA in the BTM (i.e., below the limit of detection), especially in small herds, because some infected cows may not shed *C. burnetii* in milk on the day of sampling.

Overall, RT-PCR testing of BTM using raw milk and with DMS on FTA cards showed similar results when testing was performed under the same conditions. However, when there is a delay between sampling and testing, it is preferable to store the BTM as a DMS on an FTA card to increase the probability of detecting *C. burnetii* DNA compared with performing RT-PCR on aged BTM. The use of FTA cards makes BTM sampling, shipment, and storage easy and inexpensive, and results do not seem to be impaired by the preservation and transportation method (raw BTM vs. BTM as a DMS on an FTA card). Indeed, our studies showed that the stability of *C. burnetii* DNA on an FTA card was maintained for at least 29 d at room temperature or 37°C. Therefore, this technique facilitates an easier and more practical approach to diagnosis of Q fever at the herd level and supports Q fever control strategies.
